# Clinical and Genetic Spectrum of Inborn Errors of Immunity in a Tertiary Care Center in Southern India

**DOI:** 10.1007/s12098-021-03936-w

**Published:** 2021-11-26

**Authors:** Harsha Prasada Lashkari, Manisha Madkaikar, Aparna Dalvi, Maya Gupta, Jacinta Bustamante, Madhubala Sharma, Amit Rawat, Prateek Bhatia, Kamalakshi G. Bhat, Sadashiva Rao, Nutan Kamath, Faheem Moideen, Sylvain Latour, Sarah Winter, Gandham SriLakshmi Bhavani, Katta M. Girisha

**Affiliations:** 1 Department of Pediatrics, Kasturba Medical College, Mangalore, Manipal Academy of Higher Education, Manipal, Karnataka 575001 India; 2 ICMR-National Institute of Immunohematology, KEM Hospital, Parel, Mumbai, Maharashtra India; 3 Génétique Humaine Des Maladies Infectieuses/Human Genetics of Infectious Diseases, INSERM UMR 1163, Université de Paris, Institut Imagine, Paris, France; 4 Pediatric Allergy Immunology Unit, Department of Pediatrics, Advanced Pediatrics Center, Postgraduate Institute of Medical Education & Research, Chandigarh, India; 5 Pediatric Hematology & Pediatric Molecular Hematology Laboratory, Pediatric Hematology–Oncology Unit, Department of Pediatrics, Advanced Pediatrics Center, PGIMER, Chandigarh, India; 6 Department of Pediatric Surgery, Kasturba Medical College, Mangalore, Manipal Academy of Higher Education, Manipal, Karnataka India; 7 Laboratory of Lymphocyte Activation and Susceptibility to EBV Infection, INSERM UMR 1163, Imagine Institute, Paris, France; 8 Department of Medical Genetics, Kasturba Medical College, Manipal, Manipal Academy of Higher Education, Manipal, Karnataka India

**Keywords:** Primary immunodeficiency diseases (PID), Inborn errors of immunity (IEI), Immune dysregulation, Severe combined immunodeficiency (SCID), Agammaglobulinemia

## Abstract

**Objectives:**

To study the incidence, clinical manifestations, and genetic spectrum of primary immunodeficiency diseases (PID)/inborn errors of immunity (IEI) in a tertiary care hospital in Southern India.

**Methods:**

A retrospective analysis of all patients with a clinical suspicion of PID/IEI seen at a tertiary care hospital was performed. All patients had at least one or more warning signs of PID. Serum immunoglobulin levels and other targeted investigations were performed as warranted by the clinical presentation. All families with suspected PID were counseled and offered genetic testing.

**Results:**

A total of 225 children were evaluated for PID during the study period of 6 y. Fifty-six of them did not meet the European Society of Immunodeficiencies (ESID) criteria (working definition of clinical diagnosis) and were excluded. An IEI was found in 30/49 (61.2%) patients. The most frequent reason for referral was recurrent/unusual or serious infections (28%), or cytopenia (16%). Group IV diseases of immune dysregulation was the most common category (19%), followed by group III predominant antibody deficiencies in 23/163 (14%), as per the International Union of Immunological Societies (IUIS) classification.

**Conclusions:**

This study highlights the heterogeneity of the present cohort, the underuse of genetic tests, and efforts to provide optimal care for children with possible IEI in this center.

**Supplementary Information:**

The online version contains supplementary material available at 10.1007/s12098-021-03936-w.

## Introduction

Primary immunodeficiency diseases (PID), also known as inborn errors of immunity (IEI), represent a heterogeneous group of disorders that affect the immune system, and often result in recurrent, persistent, or serious infections, autoinflammatory diseases, immune dysregulation leading to lymphoproliferation, autoimmune phenomenon, and development of cancer. The prevalence of IEI varies widely in different populations. Approximately 1 in 1,200 persons in the United States of America to 0.35 per 100,000 persons in Sweden [1–6] are estimated to be affected with an IEI. Advances in molecular genetic testing have led to the recognition of an increased number of PID and nearly 430 causative genes [7, 8]. The International Union of Immunological Societies (IUIS) expert committee categorized 406 distinct PID into ten groups based on genetic etiology [9].

PID are expected to be more prevalent in societies where consanguineous marriages are common, such as in Asian and Middle Eastern populations [10–14]. It is important to have registries of PID from different countries to understand the magnitude of the problem and to define precise management strategies. India does not have a national PID registry, and the exact prevalence of these disorders is unknown [15]. The earliest reports of PID from India were almost 50 y ago and were based on clinical manifestations rather than genetic testing [16].

In this study, the evaluation of PID and their distribution among children from coastal districts of Karnataka state are reported. This data should provide vital information to guide clinicians.

## Material and Methods

This is a retrospective study of patients with PID referred to Kasturba Medical College Hospital, Mangalore, over the last 6 y (1^st^ October 2013 to 30^th^ September 2019). The Institutional Ethics Committee (IEC KMCMLR 01/2020–70) approved the study. Clinical symptoms, signs, demographic data, age at onset, age at diagnosis, family history, consanguinity, reasons for referral, final diagnosis, and survival rates were retrieved from the medical records.

Complete hemogram, and serum immunoglobulin levels were performed in all the affected individuals. Lymphocyte subpopulations, nitroblue tetrazolium test (NBT)/dihydro rhodamine (DHR) tests were performed when indicated. Bone marrow examination, ferritin level, autoimmune workup (ANA, C3 level, direct Coombs test), anticomplement factor H antibodies, IL-12 receptor beta assay, DOCK8, WAS protein expression by flow cytometry were performed in selected patients wherever warranted by clinical judgment and affordability. Antibody response to vaccines was not assessed in any of the patients included in the study. Initial screening for PID was performed using the Jeffrey Modell Foundation’s 10 warning signs of immune deficiency [17], and was diagnosed as per the criteria laid down by the European Society of Immunodeficiency disorders (ESID) working parties registry [18]. All the affected individuals fulfilling the ESID criteria of clinical diagnosis of PID were classified using the International Union of Immunological Societies (IUIS) phenotypic classification for PID [8, 9].

All the affected individuals who met the criteria were offered a molecular testing on genomic DNA extracted from blood, which was performed either by using targeted gene sequencing panel or whole exome sequencing, based on the test availability and affordability. Sequencing of selected genes was performed in a few patients. Familial segregation was confirmed by Sanger sequencing of the parents’ samples for the disease-causing variants whenever possible. The HGVS nomenclature for the variants and American College of Medical Genetics and Genomics (ACMG) guidelines for their interpretation were followed.

## Results

Two hundred and twenty-five patients were referred to the authors’ tertiary care referral hospital of their region for evaluation of a PID. The referrals increased over time. One hundred sixty-nine children met the criteria for clinical diagnosis of a PID as per ESID (working definition of clinical diagnosis) criteria (Table 1); however, molecular genetic tests in 6 children established an alternate diagnosis. Finally, 163 children are described in this work. The most common reasons for referral were: recurrent and severe infections in 45 patients (28%), cytopenia in 26 (16%), and pyrexia of unknown origin (PUO) in 18 (11%).

**Table 1:** Patient characteristics

Patient characteristics	
Number of children referred for evaluation on PID	225
Children meeting ESID clinical criteria for PID	169
Gender	
Male	108 (64%)
Female	61 (36%)
Age at diagnosis	
< 1 y	40 (24%)
1–5 y	61 (36%)
6–10 y	34 (20%)
11–18 y	27 (16%)
> 19 y	7 (4%)
Consanguinity	
Present	40 (23%)
Absent	116 (69%)
Not known	13 (8%)
Outcome	
Alive	110 (68%)
Dead	36 (22%)
Lost to follow-up	17 (10%)
Alternative (non-PID) molecular diagnoses	6

*ESID* European Society for Immunodeficiencies; *PID* Primary immunodeficiency

Genetic testing was performed in a total of 58 children. Results are awaited for nine children while writing this manuscript. Out of 49 children, Whole exome sequencing was performed for 23 patients, gene panel testing for 12 patients, and 14 individuals underwent Sanger sequencing. An alternate genetic diagnoses were observed in six children on genetic testing: Wolman disease (1), Gaucher disease (1), Osteopetrosis (1), congenital vitamin B12 deficiency (2) and Myelodysplastic syndrome with acute myeloid leukaemia (1).

Genetic testing was nondiagnostic in thirteen children: suspected MSMD (2), hyper-IgE syndrome (2), severe congenital neutropenia (2), hemophagocytic lymphohistiocytosis (1), hyper-IgM (1), chronic granulomatous disease (1), combined immunodeficiency (1), unclassified immune dysregulation (1), C1q deficiency (1), and IL17 pathway defect (1). Details of molecular genetic test results of 30 children with diagnosis of PID are provided in Table 2.

**Table 2:** Clinical, immunological, and genetic characteristics of confirmed cases of primary immunodeficiency

Patient ID	Age/Sex	Clinical presentation	Salient immunological features	Gene (Transcript ID)	Location	Nucleotide change	Amino acid change	Zygosity	ACMG classification	Status	Disease (MIM#)	Inheritance pattern	IUIS classification 2019
P2	1 y/ Female	Multifocal tuberculosis BCG-related	LN- AFB positive hypergammaglobulinemia IL12RB1 expression-abnormal	*IL12RB1 *(NM_005535.1)	Exon 15	c.1786A>G	p.(Lys596Glu)	Homozygous	Likely benign	Novel	Immunodeficiency-30 (614891)	Autosomal recessive	Group VI
P11	7 mo/ Male	Low platelets/Eczema	Eosinophilia, low platelets, small size platelets, IgE elevated	*WAS *(NM_000377.3)	Exon 2	c.238del	p.(Gln80ArgfsTer4)	Hemizygous	Pathogenic	Known	Wiskot–Aldrich syndrome (301000)	X-linked recessive	Group IIb
P12	11 mo/ Male	Low platelets/Nephritis	Eosinophilia, low platelets, small size platelets, IgE elevated	*WAS *(NM_000377.3)	Exon 9	c.919A>G	p.(Met307Val)	Hemizygous	Variant of uncertain significance	Known	Wiskot–Aldrich syndrome (301000)	X-linked recessive	Group IIb
P25	11 y/ Female	Oral ulcer and gingival disease	Neutropenia, BMA - neutrophil maturation arrest	*ELANE *(NM_001972.4)	Exon 3	c.239T>G	p.(Val80Gl)	Heterozygous	Likely pathogenic	Known	Severe congenital neutropenia-1 (202700)	Autosomal dominant	Group V
P29	7 y/ Male	Recurrent severe infections	Neutropenia, BMA - neutrophils maturation arrest	*HAX1 *(NM_006118.3)	Exon 3	c.430delG	p.val144fs	Homozygous	Pathogenic	Known	Kostman syndrome	Autosomal recessive	Group V
P31	28 y/ Female	Recurrent/persistent oral candidiasis and onychomycosis	Ig profile - normal, LSS - normal	*STAT1 *(NM_007315.4)	Exon 10	c.821G>A	p.(Arg274Gln)	Heterozygous	Pathogenic	Known	Immunodeficiency 31C, autosomal dominant (614162)	Autosomal dominant	Group VI
P32	5 y/ Male	Recurrent/persistent oral candidiasis and onychomycosis	Ig profile - normal, LSS - normal	*STAT1 *(NM_007315.4)	Exon 10	c.821G>A	p.(Arg274Gln)	Heterozygous	Pathogenic	Known	Immunodeficiency 31C, autosomal dominant (614162)	Autosomal dominant	Group VI
P33	3 y/ Female	Recurrent/persistent oral candidiasis and onychomycosis	Not performed	*STAT1 *(NM_007315.4)	Exon 10	c.821G>A	p.(Arg274Gln)	Heterozygous	Pathogenic	Known	Immunodeficiency 31C, autosomal dominant (614162)	Autosomal dominant	Group VI
P39	6 y/ Female	Multifocal tuberculosis and candidiasis	LN -AFB positive T cells reduced IL12 RB1 expression - normal	*RORC *(NM_005060.4)	Exon 5	c.558T>G	p.(Tyr186Ter)	Homozygous	Pathogenic	Novel	Immunodeficiency 42 (616622)	Autosomal recessive	Group VI
P40	28 y/ Male	Low platelets/Eczema/Ear discharge/Nephritis/Vasculitis	Eosinophilia, low platelets, small size platelets, IgE elevated, hypogammaglobulinemia	*WAS *(NM_000377.3)	Exon 10	c.961C>T	p.(Arg321Ter)	Hemizygous	Pathogenic	Known	Wiskott–Aldrich syndrome (301000)	X-linked recessive	Group IIb
P42	8 mo/ Male	Recurrent diarrhoea with blood in stools, low platelets	Eosinophilia, low platelets, small size platelets, hypogammaglobulinemia, IgE elevated	*WAS *(NM_000377.3)	Exon 10	c.961C>T	p.(Arg321Ter)	Hemizygous	Pathogenic	Known	Wiskott–Aldrich syndrome (301000)	X-linked recessive	Group IIb
P43	3 mo/ Male	Persistent severe infection	Leukocytosis, hypergammaglobulinemia, NBT and DHR - abnormal	*CYBB *(NM_000397.4)	Exon 10	c.1234G>A	p.(Gly412Arg)	Hemizygous	Likely pathogenic	Known	X-linked chronic granulomatous disease (306400)	X-linked recessive	Group V
P57	4 y/ Female	Recurrent infections/Colitis	Leukocytosis, hypergammaglobulinemia, NBT and DHR - abnormal	*NCF1 *(NG_009078.2)	Intron 2	c.153+5G>C	-	Homozygous	Variant of uncertain significance	Known	Chronic granulomatous disease due to deficiency of NCF-1 (233700)	Autosomal recessive	Group V
P58	1 mo/ Female	PUO and history of sibling death at 6 mo	Leukocytosis, hypergammglobulinemia, NBT and DHR - abnormal	*CYBA *(NM_000101.4)	Exon 5	c.269G>A	p.(Arg90Gln)	Homozygous	Likely pathogenic	Known	Chronic granulomatous disease, autosomal, due to deficiency of CYBA (233690)	Autosomal recessive	Group V
P60	1 y/ Male	Persistent pneumonia	Leukocytosis, hypergammaglobulinemia, NBT and DHR -abnormal	*NCF1 *(NM_000265.6)	Exon 2	c.75_76delGT	p.(Tyr26HisfsTer26)	Homozygous	Pathogenic	Known	Chronic granulomatous disease due to deficiency of NCF-1 (233700)	Autosomal recessive	Group V
P61	10 y/ Male	Recurrent infections	Agammaglobulinemia abnormal, BTK expression - absent B cells	*BTK *(NM_000061.3)	Exon 17	c.1750G>A	p.(Gly584Arg)	Hemizygous	Likely pathogenic	Known	X-linked agammaglobulinemia (300755)	X-linked recessive	Group III
P63	5 y/ Male	Recurrent infections	Agammaglobulinemia abnormal, BTK expression - absent B Lymphocytes	*BTK *(NM_000061.3)	Exon 17	c.1686_1696del	p.(Trp563GlyfsTer6)	Hemizygous	Pathogenic	Novel	X-linked agammaglobulinemia (300755)	X-linked recessive	Group III
P64	4 y/ Male	Eosinophilia/Eczema/Recurrent infections	Eosinophilia, IgE - elevated	*STAT3 *(NM_139276.2)	Exon 21	c.1970A>G	p.(Tyr657Cys)	Heterozygous	Likely pathogenic	Known	Hyper-IgE recurrent infection syndrome (147060)	Autosomal dominant	Group IIb
P65	11 y/ Male	Left chest parasternal swelling and unusual type of lymphoma in a child	Lymph node biopsy -marginal zone lymphoma, EBV DNA 39000 copies, hypergammaglobulinemia, lymphopenia	*RASGRP1* (NM_005739)	Exon 8	c.941C>A	p.(Ser314*)	Homozygous	Pathogenic	Known	RASGRP1	Autosomal recessive	Group IV
P77	11 mo/ Male	Recurrent infections/Skin rashes	Anemia, eosinophilia, hypogammaglobulinemia	*IL2RG *(NM_000206.3)	Exon 5	c.670C>T	p.(Arg224Trp)	Hemizygous	Pathogenic	Known	Severe combined immunodeficiency, X-linked (300400)	X-linked recessive	Group I
P83	11 y/ Female	Cytopenia	Neutropenia, Ig profile – normal, bone marrow aspiration - maturation arrest of neutrophils	*HAX1 *(NM _006118.3)	Exon 3	c.430delG	p.val144fs	Homozygous	Pathogenic	Known	Kostman syndrome	Autosomal recessive	Group V
P85	2 y/ Male	Recurrent infections	Neutropenia, agammaglobulinemia abnormal, BTK expression - absent B cells	*BTK *(NM_000061.3)	Exon 10	c.842G>A	p.(Trp281Ter)	Hemizygous	Pathogenic	Known	X-linked agammaglobulinemia (300755)	X-linked recessive	Group III
P117	30 y/ Male	Eczema/Onychomycosis	Eosinophilia, IgE - elevated	*STAT3 *(NM_139276.2)	Exon 10	c.986T>A	p.(Met329Lys)	Heterozygous	Likely pathogenic	Novel	Hyper-IgE recurrent infection syndrome (147060)	Autosomal dominant	Group IIb
P125	11 mo/ Male	Leucocytosis/Thrombocytopenia/ PUO	Lymphocytosis, Dawney cells (reactive lymphocytes), EBV DNA PCR - > 1 lakh copies, hypergammaglobulinemia, CD4 cells - reduced, CD 8 cells - elevated	*SH2D1A *(NM_002351.4)	Exon 2	c.163C>T	p.(Arg55Ter)	Hemizygous	Pathogenic	Known	X-linked lymphoproliferative syndrome-1 (308240)	X-linked recessive	Group IV
P128	8 y/ Male	Recurrent infections	Agammaglobulinemia abnormal, BTK expression –absent B cells	*BTK *(NM_000061.3)	Exon 15	c.1445T>G	p.(Leu482Arg)	Hemizygous	Likely pathogenic	Novel	X-linked agammaglobulinemia (300755)	X-linked recessive	Group III
P129	3 y/ Male	Recurrent infections	Agammaglobulinemia abnormal, BTK expression –absent B cells	*BTK *(NM_000061.3)	Exon 16	c.1594C>T	p.(Gln532Ter)	Hemizygous	Pathogenic	Known	X-linked agammaglobulinemia (300755)	X-linked recessive	Group III
P132	2 y/ Male	Low platelets/Ear discharge	Eosinophilia, low platelets, small size platelets, IgE -elevated	*WAS *(NM_000377.3)	Exon 9	c.919A>G	p.(Met307Val)	Hemizygous	Variant of uncertain significance	Known	Wiskott–Aldrich syndrome (301000)	X-linked recessive	Group IIb
P135	2 y/ Female	Recurrent oral thrush	Immunoglobulin levels normal	*STAT1* (NM 007315.3: GOF)	Exon 14	c.1154C>T		Heterozygous	Pathogenic	Known	CMC	Autosomal dominant	Group VI
P137	3 mo/ Male	Cytopenia/PUO	Granules in neutrophils and lymphocytes, high ferritin, hemophagocytes on bone marrow aspiration	*LYST *(NM_000081.4)	Exon 9	c.3787C>T	p.(Gln1263Ter)	Homozygous	Pathogenic	Novel	Chediak–Higashi syndrome (214500)	Autosomal recessive	Group IV
P140	10 mo/ Female	Recurrent infections/Eczema	Lymphopenia, eosinophilia, IgE -elevated, low CD 4 counts	*DOCK8 *(NM_203447.4)	Exon 44	c.5625T>G	p.(Tyr1875Ter)	Homozygous	Pathogenic	Known	Hyper-IgE recurrent infection syndrome, autosomal recessive (243700)	Autosomal recessive	Group IIb

*AFB *Acid fast bacilli; *BMA* Bone marrow aspiration; *CMC* Chronic mucocutaneous candidiasis; *CNV* Copy number variations; *DHR* Dihydrorhodamine; *LN* Lymph node; *NBT* Nitroblue tetrazolium test

The breakpoint of this CNV is different to the CNV of exon 12 previously reported [22]

According to IUIS classification [19], group IV diseases of immune dysregulation were seen in 31/163 (19%) of patients and was the most common category in the present cohort, followed by group III predominant antibody deficiencies in 23/163 (14%) (Table 3). The clinical features and the tests performed in the present cohort to diagnose most frequent types of PID according to their category are described in the Supplementary material, which is available online.

**Table 3:** Diagnoses of primary immunodeficiencies as per ESID clinical criteria in the cohort (*n* = 163)

* Immunodeficiencies affecting cellular and humoral immunity*	21
(a) Severe combined immunodeficiencies SCID, defined by CD3 T cell lymphopenia.	10
(b) Combined immunodeficiencies generally less profound than severe combined immunodeficiency	12
* Combined immunodeficiency (CID)*	21
(a) CID with associated or syndromic features (congenital thrombocytopenia, DNA repair defects, thymic defects with additional congenital anomalies, immuno-osseous dysplasias)	8
(b) CID with associated or syndromic features (hyper-IgE syndrome, others)	13
* Predominantly antibody deficiencies*	23
(a) Hypogammaglobulinemia (X-linked agammaglobulinemia, common variable immunodeficiency phenotype)	20
(b) Other antibody deficiencies (hyper-IgM syndromes)	3
* Diseases of immune dysregulation*	31
(a) Hemophagocytic lymphohistiocytosis HLH & EBV susceptibility	11
(b) Syndromes with autoimmunity and immune dysregulation with colitis	20
* Congenital defects of phagocyte number, function, or both *	22
(a) Neutropenia	8
(b) Functional defects	14
* Defects in intrinsic and innate immunity *	21
(a) Bacterial and parasitic infections	8
(b) MSMD and viral infections	13
* Autoinflammatory disorders *	11
(a) Autoinflammatory disorders (recurrent inflammation, systemic inflammation with urticarial rashes, others)	10
(b) Autoinflammatory disorders (sterile inflammation of skin, bones, and joints)	1
* Complement deficiencies *	10
* Bone marrow failure *	3
* Phenocopies of PID *	0

Among the disease causing (pathogenic) variants, 5 variants out of the 25 found are novel, and the remaining (20/25) have been reported previously in the literature. The variants, p.(Leu482Arg) and p.(Trp563GlyfsTer6) of *BTK*, p.(Gln1263Ter) of *LYST*, p.(Tyr186Ter) of *RORC* and p.(Met329Lys) of *STAT3* are novel variants. These variants are not present in the gnomAD population database and are predicted to be disease causing by pathogenicity prediction software. In terms of treatment, only two patients (severe combined immunodeficiency and Wiskott–Aldrich syndrome) underwent hematopoietic stem cell transplantation (HSCT). Among 146 children at the last follow-up, 110 (75%) were alive and 36 (25%) deceased. The survival status of remaining 17 children is not known, as they lost to follow-up.

## Discussion

In this study, clinical, immunological, and genetic characteristics of a cohort of patients referred for evaluation for an IEI is described. The present study showed that the patients displayed recurrent infections, cytopenia, PUO, eczema, lymphadenopathy, recurrent oral thrush, or multifocal tuberculosis as the main reasons for their referral. It is interesting to note that 13 out of 29 children with identified rare variants in known IEI-causing genes were males with hemizygous variants and X-linked recessive conditions. In this study, 23% of the patients were born to consanguineous parents. The reported rate of consanguinity in India varies between 30% [16] to 80% [20]. In contrast, the United Kingdom PID registry recorded consanguinity only in 2.9% patients [2]. Rare variants of IEI-disease causing genes were found in 19% of the patients who underwent genetic studies. The UK PID registry reported 21.8% of patients with a proven genetic defect underlying their PID.

The three most common reasons for referral accounting for 49% were: recurrent fever, cytopenia, and PUO. Verma et al. published a series of 27 children with PID and the typical presentation was recurrent or persistent chest infection [21]. In contrast to the present report, Gupta et al. showed that lower respiratory tract infections, gastrointestinal illnesses such as diarrhea, and failure to thrive were the common symptoms leading to the diagnosis of a PID in a cohort of 120 children from north India [20]. These differences may be due to regional differences, and level of awareness among the referring clinicians and even a reflection of inbreeding practices. The authors unit predominantly caters to hematology and oncology; hence, this might have also caused the difference in the spectrum of IEIs.

The most common category noted in the present cohort was group IV diseases of immune dysregulation seen in 19% of patients. Syndromes with autoimmunity and immunodeficiency with colitis were the most common subtypes within this group. The group III disorders (predominantly antibody deficiencies) were seen in 14%, followed by group V (congenital phagocytic disorders) in 13.4%. Group I (immunodeficiencies affecting humoral and cellular immunity) and group II (combined immunodeficiency with syndromic features) were noted in 12.8% of patients each. The pattern of PID in the present study is different from other published studies from India. Madkaikar et al. [15] reported that phagocytic dysfunction and immune dysregulations were the two most common PID categories [14]. A study from Delhi showed congenital phagocytic disorders as the commonest category [21]. However, reports from Sri Lanka suggested antibody deficiencies (40%) to be the most frequent, followed by combined immunodeficiency with well-defined syndromes and SCID. PID registries in the UK or Europe revealed that antibody disorders represent the largest group of all registered patients, accounting for 59.7% [1, 2]. The other most frequently reported PID in western countries is the common variable immunodeficiency (CVID), accounting for 29.7% patients in the UK [2]. The present study found 14% of patients with antibody deficiency and only 1 child with CVID. No patient with selective IgA deficiency was found either in the present group or in the other studies from India. This is probably because of the benign nature of the disease. The reason for seeing more children with combined immunodeficiencies with syndromic features in the present study may be due to a referral/recruitment bias. There is an increase in PID awareness and recognition, as an increased number of patient referrals for evaluation of IEI/PID has been noted over the last six years.

Of the 29 disease-causing variants identified, 18 variants are classified as pathogenic, 8 as likely pathogenic, 2 as variants of uncertain significance, and 1 as likely benign. Despite the variant, p.(Lys596Glu) of *IL12RB1* is classified as a likely benign variant, as it is known for its low penetrance and the patient's phenotype suggests susceptibility to mycobacterial disease, this is considered as a plausible variant. However, further functional studies are required to confirm its disease-causing impact. It is also interesting to note that 6 children who had clinical features of a PID had alternate diagnoses on genetic testing. This highlights the diverse and overlapping clinical manifestations of PID with other diseases in children.

## Limitations

The present study is limited by its retrospective nature. Genetic tests could be performed in only 34% of the patients in the study. The symptom interval and the overall time taken to diagnose IEI has not been studied in the present cohort. There may be a referral bias, as the authors’ center is also the referral center for pediatric hematology. Many children died before their molecular testing, though they were meeting the clinical criteria for PID. Due to logistic issues, any functional studies could not be performed. A follow-up for 17 children could not be performed either. The total time for a genetic diagnosis has not been calculated, which is important to improve the outcome. Despite these limitations, the authors believe that their study highlights the importance of such a practice in centers like theirs and this should alert policymakers and health entrepreneurs.

An in-house flow cytometry for diagnosis of PID and hematopoietic stem cell transplant (HSCT) facility for treatment are not available with the authors. This has likely affected patients’ care and no child with SCID survived in the present cohort. This also underscores the need to have better diagnostic and HSCT facilities locally all over the country.

## Conclusions

This study reveals that recurrent infections, persistent low WBC/platelet count (cytopenia), PUO, persistent diarrhea, prolonged illness, lymphadenopathy, multifocal tuberculosis, multiple BCG adenitis, unusual lymphomas, abscesses, persistent or recurrent oral thrush, difficult eczema, atypical hemolytic uremic syndrome (HUS) are presenting symptoms of PID. Complete hemogram, counts of neutrophils, lymphocytes, and eosinophils, examination of peripheral smear, and thymic shadow on radiographs give useful clues for the diagnosis of PID. Over the past few years, increased awareness about PID among the physicians has led to an increased recognition of these conditions. However, genetic testing facilities are underused. Further prospective studies and the establishment of a nationwide PID registry will help us retrieve more information about the common categories of PID, better approaches for the diagnosis to lead to a better outcome for the children with PID.

## Supplementary Information

Below is the link to the electronic supplementary material.

Supplementary file1 (DOCX 35 KB)
