# Supplementary material for: Clinical and Genetic Spectrum of Inborn Errors of Immunity in a Tertiary Care Center in Southern India
Source: Indian J Pediatr. 2021 Nov 26;89(3):233–42. doi: 10.1007/s12098-021-03936-w (PMC8857151; doi:10.1007/s12098-021-03936-w)
Supplement: Supplementary file 1 — Supplementary file1 (DOCX 35 KB) [file 12098_2021_3936_MOESM1_ESM.docx]

**Supplementary material**

**Clinical and genetic spectrum of inborn errors of immunity in a tertiary care center in Southern India**

**Group 1: Immunodeficiencies affecting cellular and humoral immunity (*n* = 21)**

**Severe combined immunodeficiency (SCID)**

A total of 10 children (aged 7 d to 15 mo) were diagnosed with SCID. Nine of them were infants and remaining one was 15 mo old boy. They were referred with recurrent infections, Cytopenia, pyrexia of unknown origin (PUO), Eczematous rash with Omenn's syndrome phenotype, tubercular meningitis and pulmonary tuberculosis (3 mo), persistent diarrhoea and one child with failure to thrive. Barring three patients, all had low absolute lymphocyte count for the age [1, 2]. Thymic shadow was absent in 6 /10 children. Lymphocyte subsets (LSS) showed T–B+NK+ in 3 patients, two infants each with T- B+ NK- and T-B-NK- subsets. One child had a mutation in the IL2 RG gene diagnosed a few weeks after death and his brother also died at 10 mo of age with severe infection (both of them did not have lymphocyte subsets estimation). Unfortunately, all of them died. One underwent HSCT, however, died a few months later with an infection.

The remaining 11 patients were diagnosed to have combined immunodeficiency (CID) generally less profound than severe combined immunodeficiency. Three of them had low CD4 counts, two children with high IgE levels and diagnosed to have DOCK 8 deficiency and one adolescent boy developed Listeria monocytogenes meningitis. Another child had a life-threatening infection with low CD8 counts suspecting ZAP 70 defect. The remaining seven children met the ESID clinical criteria of CID but not had a specific PID diagnosis. Of the two patients diagnosed with DOCK 8 deficiency, one presented at 11 mo with generalized disseminated persistent warts and another with eczema and recurrent respiratory infections at 6 mo. Both children had high Immunoglobulin E levels of 5895IU/mL and 6340IU/mL respectively. The LSS was performed on the second child showed low lymphocyte count and abnormal DOCK 8 expression. was. In this patient, *DOCK8* (NM_203447.4) c.5625T>Gp. (Tyr1875Ter) the homozygous mutation was found. Both children with DOCK8 deficiency passed away within 6 mo of diagnosis due to respiratory tract infections.

**Group II: CID with associated or syndromic features (*n* = 21)**

**Hyper IgE (*n* = 13)**

A common reason for referral in this group of 13 children were eczema, eosinophilia, recurrent infections, and failure to thrive. Oral thrush, eczema, onychomycosis, episode of pneumonia, abnormal facies, NIH score>40 [3] noted in three patients, and high IgE levels (range 2300 to > 18000 IU/L) helped us to diagnose Hyper IgE syndrome. Three patients have had genetic tests and two had mutations in STAT 3 gene (Table 2).

**Wiskott–Aldrich syndrome (WAS) (*n* = 7)**

Wiskott Aldrich Syndrome was diagnosed in seven patients including a 34-y man. All of them were referred for evaluation of low platelet count (< 50000cells/cm^3^) when they presented with petechial rashes and eczema. Two children had features of colitis and manifestations of vasculitis. The platelet size was small in all (Mean Platelet Volume ranging 5-6fL). The 34-y-old man was diagnosed to have WAS following the diagnosis of his nephew with WAS, though he had low platelet counts since childhood and was receiving treatment as Immune thrombocytopenia. All of our patients had high IgE levels (800-2960IU/mL). The available genetic results showed a mutation in WAS gene exon 2, 9, and 10 (Table 2). One child underwent haploidentical hematopoietic stem cell transplant and is doing well. Two children with WAS died due to severe infections (one viral pneumonia and another with staphylococcus aureus sepsis). The rest four children with WAS are on regular medical supervision. The remaining one child was diagnosed with Ataxia telangiectasia (*n* = 1).

**Group III: Predominantly antibody deficiencies (*n* = 23)**

**X-Linked agammaglobulinemia (XLA) (*n* = 6)**

Six children were diagnosed to have X-linked agammaglobulinemia. They were all referred because of recurrent infections (5) and one with recurrent pneumonia. All have had at least one episode of hospitalisation and acute otitis media. One child had glomerulonephritis, septic arthritis, and low B cells (CD 19- 8.31%). He was found to have variant c.1445T>G, p. L482R in BTK gene confirming the diagnosis of XLA. His CD14 monocytes showed normal expression of BTK, however, the intensity of expression was significantly lower than the normal control. The rest 4 children had abnormal BTK protein expression on flow cytometry and CD 19 cells were < 1%. Five of them did not have any tonsils whereas the one had a small tonsil. One child also had neutropenia during viral upper respiratory tract infection. One child died due to septic shock soon after diagnosis. Rest all of them are on regular immunoglobulin replacement therapy. All except one had confirmed mutations in exons 10, 15, 16, and 17 of the BTK gene as shown in Table 2.

The remaining patients in this group were hypogammaglobulinemia (*n* = 12) who were referred with severe/recurrent infections and found to have lower IgG levels, Hyper IgM (*n* = 3), one each with Common Variable Immunodeficiency (CVID), and Autosomal recessive agammaglobulinemia. All these children are alive at the last follow up except the one with AR agammaglobulinemia who died due to respiratory infection.

**IV: Diseases of immune dysregulation (*n* = 31)**

**Hemophagocytic lymphohistiocytosis (HLH) and susceptibility to EBV infection**

Out of 31 children falling under this category of disease of immune dysregulation in our cohort, 10 were diagnosed to have HLH. All of them were less than 5 y age and were referred because of PUO (3), lymphadenopathy (2), suspected HLH in 3 (organomegaly, high-grade fever spikes, high ferritin >3500ng/ml) patients and one 6-mo-old child was referred for prolonged chickenpox infection with organomegaly and another one with cytopenia. All children met the criteria of HLH 2004 [4]. One child did have albinism, granules in lymphocytes, and neutrophils on the peripheral smear, and exome sequencing showed a mutation in the LYST gene suggestive of Chediak-Higashi syndrome [5]. The other one who was referred with leucocytosis (WBC 56000cells/cm^3^), anemia (Hb 5.5gm%), and thrombocytopenia (33000cell/cm^3^), the peripheral smear was suggestive of atypical lymphocytes with indented cytoplasm and nucleoli. The EBV DNA were > 100000 copies/mL. Exome sequence showed a mutation in the SH2D1A gene suggestive of X-linked lymphoproliferative syndrome [6]. Another boy diagnosed with marginal zone lymphoma (seen in immunocompromised children) of the left parasternal region at 10 y of age found to have low T cells, (CD3+ve) and high EBV DNA PCR copies of 39000/mL. His genetic test showed a homozygous mutation in the RASGRP1 gene [7]. One child met the criteria for Harmansky Pudlak syndrome. Of the 10 children with HLH, six children were alive at the last follow up. Remaining three children died and in one lost to follow up.

**Autoimmune lymphoproliferative syndrome (ALPS) (*n* = 5)**

Among 5 children in this group, two children presented with cytopenia and splenomegaly (low platelets 47000 cells/cm^3^) and one each with Coombs positive autoimmune hemolytic anemia, persistent lymphadenopathy not improving with antitubercular medication for 6 mo, Evans syndrome (Autoimmune hemolytic anemia and thrombocytopenia) for the past 8 y and remaining one child with leucocytosis with a white blood cell count of 82340cells/cm^3^. The vitamin B12 levels >2000ng/ml in two children and it were 1450ng/ml, 778ng/ml, 960 ng/ml (normal range 195-690ng/ml) in the other three children. The double negative T (DNT) cells (alpha-beta positive CD3+, CD4-and CD8-) were found to be increased in two children (3.5 % to 17% gated on CD 3+ cells). The remaining one child who did not have DNT cells estimated had high ALC of 37000 cells/cm^3^ positive DCT, lymphoproliferative features on the bone marrow and lymph node biopsy with no evidence of acute lymphoblastic leukaemia. Four of them are alive at the last follow up. The other diagnoses noted in this category were Unclassified immune dysregulation (n=13), ALPS like (1), IPEX (Immune dysregulation, polyendocrinopathy, enteropathy, X-linked) like syndrome (n-1), and LRBA (lipopolysaccharide (LPS)-responsive and beige-like anchor protein) in one child. Four of the thirteen children with unclassified immune dysregulation have died and one child lost to follow up. The rest were alive at the last follow up.

**V Congenital defect in phagocytic dysfunction (*n* = 22)**

**Chronic granulomatous diseases (CGD)**

Among the 21 children under the category Phagocytic defects, 10 were diagnosed to have CGD. They were referred with recurrent infections (5), PUO (4), and one child with lung abscess. All had neutrophilic leucocytosis and hypergammaglobulinemia, the NBT and DHR were abnormal. One little girl presented on day 7 of life with PUO and was found to have a perianal abscess. One patient was diagnosed at 26 y of age who started to have symptoms from the first year of life and was hospitalised more than 30 times. Over the time, he had developed colitis and bronchiectasis. He received anti tubercular medication on 3 separate occasions for 6 mo each time. The results of genetic tests performed on 4 patients showed an autosomal recessive mutation in CYBA (1), NCF1 (2), and one X-linked recessive mutation in the CYBB gene (Table 2). All children diagnosed to have CGD died due to infections except one who underwent a successful HSCT.

The remaining patients in this group were diagnosed to have SCN (Severe Congenital Neutropenia n=7, GATA2 deficiency (n=2)), LAD- (Leukocyte Adhesion Defect n=1) and one child with unclassified phagocytic dysfunction who has had recurrent abscess with neutrophilic leucocytosis, hypergammaglobulinemia and normal NBT test. One child with SCN died in the neonatal age group, one lost to follow up, and the remaining five children were alive at last follow up. Four of them on daily Granulocyte colony-stimulating factor (G CSF) injections. The one suspected GATA2 deficiency child who had recurrent abscesses and Myelodysplastic syndrome (MDS) also died.

**Group VI: Defects in intrinsic and innate immunity (*n* = 21)**

**Mendelian susceptibility of mycobacterial tuberculosis (MSMD)**

A total of 13 patients were diagnosed in this group. Six of them were referred with a history of multifocal tuberculosis (skeletal + lymph node, abdominal + neurological, lungs + lymph nodes or disseminated tuberculosis), and another five children for evaluation multiple lymphadenitis following BCG (Bacillus Calmette Guerin) vaccination. The remaining two patients were referred because of numerous lymph node swellings with sinus formation and persistent lymph node tuberculosis on therapy for a year. Two of them had low IL12 Receptor beta expression. One had a mutation in the IL12 RB1 gene. One girl presented with psoas abscess, which was found to be due to tuberculosis, and later developed seizures due to multiple tuberculoma in the brain for evaluation. She had a reduced number of T cells for the age. Her genetic test showed biallelic RORc mutation [8]. The immunoglobulin levels and T, B, and NK cell estimation were normal in all 12 children. One child and young adult due to disease progression and the other one lost to follow up. Rest are all alive at the last follow up.

**Chronic mucocutaneous candidiasis (CMC)**

A total of 6 patients were diagnosed with CMC when presented with recurrent oral thrush, fungal nail infection. Three members from one family (mother and her two children) were suffering from a sore mouth with poor quality of life. All of them had STAT1 Gain of Function mutation in exon 10 [9]. The other family who had three affected members one of the genetic reports available and showed a mutation in STA1 exon 14. Four of them were on regular antifungal therapy and the remaining two were on antifungal prophylaxis.

The remaining one patient in this category was diagnosed to have hydradenitis suppurativa.

**VII: Autoinflammatory disorders (*n* = 11)**

A total of 11 children were diagnosed to have the auto-inflammatory syndrome. They all presented with recurrent fever (2), PUO (8), and leukocytosis (1). All of them had prolonged fever for more than 3 weeks and all the infective workup blood culture, urine culture, tuberculosis work up including Mantoux test, chest x-ray, Cartridge based nucleic acid amplification technique (CBNATT) on gastric aspirate (6) lymph node biopsy (4), were negative. The autoimmune (Antinuclear antibody, C3 level) workup was negative in all patients. All of them underwent a bone marrow examination, which was normal. The CBC in all patients showed neutrophilic leucocytosis and platelets were more than 450000 cells/cm^3^. The inflammatory markers such as CRP (30-150mg/L) and ESR (40-86mm/hr) were high. All these children were alive at the last follow up.

**VIII: Complement deficiencies (*n* = 10)**

Seven of ten children in this group were referred to our centre for evaluation of atypical hemolytic uremic syndrome (HUS). They presented with hemolysis, low platelets, unconjugated jaundice and acute kidney injury (AKI) with no prior history of gastroenteritis. Autoimmune workup (ANA) was negative and C3 was in the normal range in all patients. The antibodies to complement factor H levels were high between 260-1565 AU/mL (Normal < 150AU/mL) in all seven children. One of the remaining three children with complement deficiencies was referred with repeated episodes of swelling of lips and face and abdominal pain. Her C4 levels were low and suggestive of hereditary angioedema. The other one was referred with cytopenia and features of lupus at 3 y of age with abnormal CH 50 assay diagnosed to have C1q deficiency. The child diagnosed with unclassified complement deficiencies presented with recurrent bacterial meningitis and CH50 assay was abnormal. Of the seven children with atypical HUS, five have died due to renal failure. The remaining two are on follow up. The child with unclassified complement deficiencies is on follow up with prophylactic antibiotics.

**IX: Bone marrow failure (*n* = 3)**

Three children with Dyskeratosis Congenita were included in this category of which two children died due to severe infections. They were referred to us for evaluation of pancytopenia.

**References**

1. Narula G, Khodaiji S, Bableshwar A, Bindra M. Age-related reference intervals for immunoglobulin levels and lymphocyte subsets in Indian children. Indian J Pathol Microbiol. 2017;60:360–4.

2. Tosato F, Bucciol G, Pantano G, et al. Lymphocytes subsets reference values in childhood. Cytom Part A. 2015;87:81–5.

3. Schimke LF, Sawalle-Belohradsky J, Roesler J, et al. Diagnostic approach to the hyper-IgE syndromes: Immunologic and clinical key findings to differentiate hyper-IgE syndromes from atopic dermatitis. J Allergy Clin Immunol. 2010;126:611–7.

4. Henter JI, Horne A, Aricó M, et al. HLH-2004: Diagnostic and therapeutic guidelines for hemophagocytic lymphohistiocytosis. Pediatr. Blood Cancer. 2007;48:124–31.

5. Rodina YA, Matveev VE, Balashov DN, Dubrovina ME, Shcherbina AY. Chediak-Higashi syndrome. Pediatr Hematol. 2016;15,27–33.

6. Morra M, Howie D, Grande MS, et al. X-linked lymphoproliferative disease: a progressive immunodeficiency. Annu Rev Immunol. 2001;19:657–82.

7. Winter S, Martin E, Boutboul D, et al. Loss of RASGRP 1 in humans impairs T‐cell expansion leading to Epstein‐Barr virus susceptibility . EMBO Mol Med. 2018;10:188–99.

8. Okada S, Markle JG, Deenick EK, et al. Impairment of immunity to candida and mycobacterium in humans with bi-allelic RORC mutations. Science. 2015;349:606–13.

9. Toubiana J, Okada S, Hiller J, et al; International STAT1 Gain-of-Function Study Group.  Heterozygous STAT1 gain-of-function mutations underlie an unexpectedly broad clinical phenotype. Blood. 2016;127:3154–64.
